# Stratified Community Responses to Methane and Sulfate Supplies in Mud Volcano Deposits: Insights from an *In Vitro* Experiment

**DOI:** 10.1371/journal.pone.0113004

**Published:** 2014-11-13

**Authors:** Yu Zhang, Lois Maignien, Alina Stadnitskaia, Pascal Boeckx, Xiang Xiao, Nico Boon

**Affiliations:** 1 State Key Laboratory of Microbial Metabolism, State Key Laboratory of Ocean Engineering, Shanghai Jiao Tong University, Shanghai, People's Republic of China; 2 Laboratory of Microbial Ecology and Technology, Ghent University, Gent, Belgium; 3 Department of Marine Geology and Chemical Oceanography, Royal Netherlands Institute for Sea Research, Texel, the Netherlands; 4 Laboratory of Applied Physical Chemistry, Ghent University, Gent, Belgium; University of Milan, Italy

## Abstract

Numerous studies on marine prokaryotic communities have postulated that a process of anaerobic oxidation of methane (AOM) coupled with sulfate reduction (SR) is the main methane sink in the world's oceans. AOM has also been reported in the deep biosphere. But the responses of the primary microbial players in eliciting changes in geochemical environments, specifically in methane and sulfate supplies, have yet to be fully elucidated. Marine mud volcanoes (MVs) expel a complex fluid mixture of which methane is the primary component, forming an environment in which AOM is a common phenomenon. In this context, we attempted to identify how the prokaryotic community would respond to changes in methane and sulfate intensities, which often occur in MV environments in the form of eruptions, diffusions or seepage. We applied an integrated approach, including (i) biochemical surveys of pore water originated from MV, (ii) *in vitro* incubation of mud breccia, and (iii) prokaryotic community structure analysis. Two distinct AOM regions were clearly detected. One is related to the sulfate methane transition zone (SMTZ) at depth of 30–55 cm below the sea floor (bsf); the second is at 165–205 cm bsf with ten times higher rates of AOM and SR. This finding contrasts with the sulfide concentrations in pore waters and supports the suggestion that potential AOM activity below the SMTZ might be an important methane sink that is largely ignored or underestimated in oceanic methane budget calculations. Moreover, the incubation conditions below the SMTZ favor the growth of methanotrophic archaeal group ANME-2 compared to ANME-1, and promote the rapid growth and high diversity of bacterial communities. These incubation conditions also promote the increase of richness in bacterial communities. Our results provide direct evidence of the mechanisms by which deep AOM processes can affect carbon cycling in the deep biosphere and global methane biochemistry.

## Introduction

The existence of microbial life in the deep subsurface has been known since ZoBell's studies in the 1930s [1] and was first proven in sediment cores during drilling in the 1980s [2]. However, whether the deep biosphere is the largest prokaryotic habitat on Earth is an enigma because the estimations of cell numbers and biomass differ dramatically among sampling sites and counting techniques [3]–[5]. The importance of these deeply buried communities for driving carbon and nutrient cycling and for catalyzing a multitude of reactions among rocks, sediment and fluids is widely accepted [6]. Available reports demonstrate that the highest quantities of active prokaryotes are associated with diverse biogeochemical interfaces, e.g., highly organic rich sediments such as the Mediterranean sapropels [7]; the sulfate methane transition zone (SMTZ), where anaerobic methanotrophy is the driving force behind local microbial activities [4]; the deep hypersaline anoxic lakes, where the ecosystems are largely driven by sulfur cycling and methanogenesis [8]; an increase in microbial biomass was also observed in sediments with gas hydrates [9].

Recent developments in research on the marine methane cycle have shown that the anaerobic oxidation of methane (AOM) is the key microbial action responsible for methane turnover in the ocean and is the first step in making energy available to the local ecosystem [10]. The AOM process has been proposed to involve reverse methanogenesis [11], [12], which is coupled with sulfate reduction via anaerobic methanotrophic archaea (ANME) and diverse sulfate-reducing bacteria (SRB) [13], [14]. Because ANME and SRB annually consume approximately 85% of oceanic methane production, the assessment of *in situ* AOM rates plays a vital role in global methane budget modeling [10]. Currently, estimations of *in situ* AOM rates are primarily based on the methane/sulfate turnover rates using radioactive tracers. Long-term incubation under simulated conditions has not been frequently reported and has only been performed at shallow sediment depths near the SMTZ [10].

Marine mud volcanoes (MVs) are among the most spectacular seepage-related geomorphological structures and produce a strong outburst of methane-saturated geofluids from the deep subsurface. The development of a MV is related to strong lateral or vertical compressions of the Earth's crust that provoke deep-lying sediments to move upward [15]. Such emitted sedimentary material is called “mud breccia” and represents exclusively MV-related deposits [16]. The main gaseous component in the seeping fluids is methane, a strong greenhouse gas. In addition to methane, mixtures of wet gas, hydrogen sulfide, carbon dioxide, and petroleum products are often present. Such chemically complex allochthonous sedimentary and fluid mixtures incite the development of particular environments at and below the sea floor and fuel microbial processes that shape the community structure of the chemosynthetic seepage.

**Figure 1:**
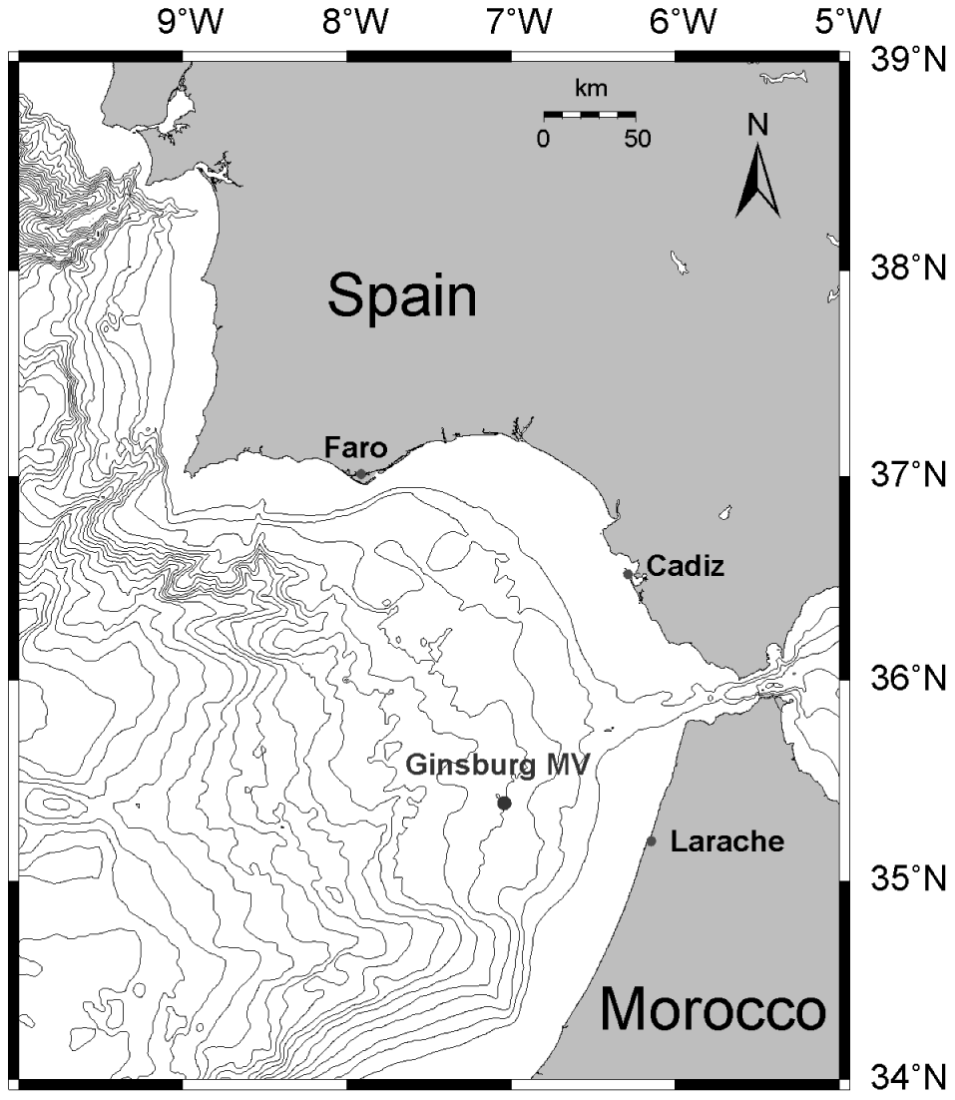
Geological map indicating the location of the Ginsburg MV based on GEBCO (General Bathymetric Chart of the Oceans) bathymetry. The depth difference between two contour lines is 250 m; the closest contour line to the coast represents 250 m water depth.

MVs are known to exhibit environmental heterogeneity, which is directly related to the mode of MV eruptions and to the chemistry of the expelled products. Hydrocarbon-rich fluids expelled to the surface bring up methane, which is utilized as a carbon and energy source. Because AOM is the initial step in biological energy conversion within the local ecosystem, the bioavailability of methane directly determines energy supply and the biomass in the sediment along the fluid migration pathway. Additionally, microbial AOM activity controls the storage of methane in the ocean. However, the distribution of active methane-consuming microbes, especially those that perform anaerobic methanotrophy along the sedimentary section of a MV, has yet to be sufficiently investigated. Furthermore, the organization of prokaryotic communities and their structure at varying sedimentary depths and in environments with variable methane concentrations has not been reported thus far. To test the potential for *in situ* AOM activity, we report the results of an *in vitro* incubation experiment under methane- and sulfate-rich conditions that was performed on freshly recovered mud breccia from the Ginsburg MV in the Gulf of Cadiz (Fig. 1). The availability of freshly erupted MV deposits allowed us to examine potential AOM activity at and below the SMTZ. We applied a vertical profile sampling strategy to reveal changes in AOM community structure and its spatial distribution at varying depths and to identify possible ecological factors that influence the community's metabolic behavior and dynamics.

## Materials and Methods

### Sampling site location and lithology

Sampling location is Ginsburg MV (35°22.431′N; 07°05.291′W) in the Gulf of Cadiz. The field studies did not involve endangered or protected species and no specific permissions were required for these locations/activities. The Gulf of Cadiz is an extensive embayment of the Atlantic Ocean from Cape Saint Vincent, Portugal, to the Gibraltar Strait on the southwestern coast of the Iberian Peninsula (Fig. 1). The area is known for its MVs [17]–[21], and the Ginsburg MV is located within the Western Moroccan MV Field of the gulf. A gravity core was taken and in total 3.55 m long of mud breccia was recovered from the crater of the MV (water depth ca. 910 m; core M2007-56) during a MicroSYSTEMS cruise of R/V Pelagia in 2007. The sampling location was the same one from which cores were collected during the TTR-9 (1999) and TTR-10 (2000) cruises undertaken for hydrocarbon gas and lipid biomarker studies [22], [23]. The lithology, presence of chemosynthetic tube worms at the surface layer and gas hydrates at depths below 1 m of sediment provided a mirror image of the previously recovered MV deposits taken during several TTR cruises [17], [19]–[21].

### Pore water sampling

The recovered sedimentary core was immediately cut into 1 m sections and opened in the cold lab container at +4°C for sub-sampling. Pore water samples were obtained on board at +4°C directly from mud breccia using a Rhizon Core Solution Sampler (Rhizosphere Research Products, Wageningen, the Netherlands). The sampler consisted of a 10 cm porous polymer tube, which was impermeable to bacteria to maintain the sterility of the samples, connected to a 10 cm PVC tube and a Luer-Lock connector attached to the standard 10 ml syringe. By drawing the piston, filtered pore waters were collected in the syringe in the vacuum.

To assess sulfate levels, 1 ml of pore water was placed in a plastic vial and kept at −20°C. To distinguish stable carbon isotopes from dissolved inorganic carbon (δ^13^C-DIC), 2 ml of pore water was preserved with 10 µl of saturated mercury chlorite solution and stored in darkness at +4°C in gas-tight glass vials without head space. Sulfide was preserved in 5 ml glass vials containing 0.5 ml of pore water and 4.5 ml of 0.1 N NaOH solution, which was initially refluxed with N_2_ for 5 min. Samples with preserved H_2_S were stored at +4°C.

### *In vitro* incubation experiment

For the incubation experiment, mud breccia samples were collected directly at 4°C. Sub-sampling was carried out along the total length of the core at 5 cm intervals using 50 ml plastic sterile syringes with cut tips. Samples were immediately sealed in trilaminate PEI aluminum bags (KENOSHA C.V., Amstelveen, the Netherlands) in a nitrogenous atmosphere and stored at +4°C until use.

In off-shore laboratories, the experiments were carried in an anoxic N_2_ atmosphere in a glove box (Concept 1000, L.E.D. Techno NV, Belgium). In total, 26 sections along the 2.55 m of the mud breccia section were selected. From each layer, 30 ml of fresh mud volcano deposits were diluted 5 times with artificial seawater medium in a 250 ml Scott bottle closed with a gas-tight butyl stopper. The headspace was first flushed and filled with ^12^C-CH_4_ at 0.1 MPa of absolute pressure (the sum of gauge pressure and atmospheric pressure) and then pressurized up to 0.15 MPa of absolute pressure by ^13^C-CH_4_ (99% Atom, Campro Scientific GmbH, Berlin, Germany). The Scott bottles were placed on a shaker (80 rpm shaking) at 15°C in darkness. The total duration of the experiment was 176 days.

### Chemical analysis of the incubated samples

Slurry samples were taken after days 8, 21, 31, 45, 73, 102, 135, and 176 of incubation. At each selected interval, approximately 3 ml of slurry was collected through a syringe without open the bottle, completely filled into two 1.5 ml Eppendorf tubes (Eppendorf, Hamburg, Germany), and centrifuged at 13,200 *g* for 2 min. The supernatant was collected and used for the determination of dissolved sulfide, sulfate and pH according to a previously described method [24]. The residue (∼1.3 ml in each tube) was frozen at −20°C for future DNA extraction.

Gas analysis was performed on samples incubated for 8 and 176 days. The gas pressure inside the incubation bottle was measured with an INFIELD 7C Tensiometer (UMS, München, Germany). From each incubation bottle, 0.5 ml of gas was removed and immediately injected into a 12 ml vacuumed gas sampling tube (Labco Limited, Buckinghamshire, UK). Next, the gas sampling tube was filled with helium up to atmospheric pressure. The CO_2_ concentration (including both ^13^C-CO_2_ and ^12^C-CO_2_) was quantified using a gas chromatograph (GC 14B, Shimadzu Corporation, Kyoto, Japan) equipped with a 2 m Porapak Q column (0.3 cm o.d., SS 80/100) and a pre-column (1 m) of the same material (both at 35°C) and a ^63^Ni electron capture detector (ECD) at 250°C. The ratio between ^13^C-CO_2_ and ^12^C-CO_2_ was quantified with an isotope ratio mass spectrometer (IRMS 20-20, Sercon Ltd, Cheshire, UK) coupled to a GC in a climatized room (21.0±0.5°C) using the same technical settings as described previously [25].

### *In vitro* AOM and SR rate calculations

Both AOM and SR rates were expressed as nmol sulfide/CO_2_ production per ml of fresh sediment per day (nmol/ml rs/d). The turnover rate was calculated according to the following formula:
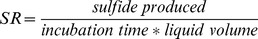
For the AOM rate calculation, the total production of ^13^C-carbon species, i.e., ^13^C-CO_2_ in both liquid and gas phases, ^13^C-HCO_3_^−^ and ^13^C-H_2_CO_3_ in liquid phase, was first calculated. Given that 1/3 of the initial headspace consisted of ^13^C-CO_2_ and 2/3 consisted of ^12^C-CO_2_, the overall AOM rate was calculated as follows:
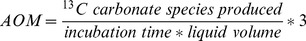



### Prokaryotic community analysis of the incubated samples

#### DNA extraction

DNA extraction was performed on the samples taken on day 0 and day 176 at each sediment depth, in total 52 samples. The residue after chemical analysis was thaw and mixed, from which a 0.5 ml slurry was used to extract DNA using Fast DNA Spin Kit for soil (Bio 101, Q-Biogene, Heidelberg, Germany) according to the manual supplied with the kit. The raw DNA was then purified with a DNA Purification Kit (Wizard, Promega, Madison, USA) and eluted to a final volume of 50 µl.

#### Terminal restriction fragment length polymorphism (T-RFLP) of the prokaryotic community

To amplify bacteria, the primers 27f-FAM and 907r (Table 1) were used under the condition described in Table S1 and Table S2. The obtained PCR product was purified with a PCR purification kit (Qiagen, Hilden, Germany) and eluted to a final volume of 30 µl. The DNA concentration in the PCR products was quantified using a NanoDrop ND-1000 Spectrophotometer (Thermo Scientific, Wilmington, USA). Then, 100 ng of the purified PCR product was added to Tango buffer (Fermentas, Burlington, Canada) and digested with 2.5 units of restriction enzyme Mspl at 37°C for 3 hours. After digestion, 100 µl cold ethanol (95%) was added, and the sample was incubated at 4°C for 30 min to precipitate the digested DNA fragments. Subsequently, the samples were centrifuged at 14,000 *g* for 30 min. The pellet obtained from the precipitation was further washed with 100 µl cold ethanol (75%) and centrifuged at 14,000 *g* for 10 min. The supernatant was discharged, and the pellet was vacuum dried for 5 min using Savant SpeedVac DNA 110 (GMI, Minnesota, USA).

**Table 1:** Primers and probes used in this study.

Name (labeling)	Sequence (5′ to 3′)	Specificity	References
primer			
Arch-21f	TTC CGG TTG ATC CYG CCG GA	Archaea	[37]
Arch-21f-FAM	FAM-TTC CGG TTG ATC CYG CCG GA	Archaea	Modified from [37]
27f-FAM	6-FAM-AGA GTT TGA TCC TGG CTC AG	Bacteria	[38]
907r	CCG TCA ATT CCT TTR AGT TT	Bacteria	[38]
Arch-958r	YCC GGC GTT GAM TCC AAT T	Archaea	[37]
Uni-1392r	ACG GGC GGT GTG TRC	Universal	[39]
probe			
ANME1-350	AGT TTT CGC GCC TGA TGC	ANME-1 archaea	[40]
EelMS932	AGC TCC ACC CGT TGT AGT	ANME-2 archaea	[40]
ANME3-1249	TCG GAG TAG GGA CCC ATT	ANME-3 archaea	[41]
ANME3-1249H3	GTC CCA ATC ATT GTA GCC GGC	Helper probe for ANME3-1249	[42]
ANME3-1249H5	TTA TGA GAT TAC CAT CTC CTT	Helper probe for ANME3-1249	[42]
DSS658	TCC ACT TCC CTC TCC CAT	*Desulfosarcina* spp., *Desulfofaba* spp., *Desulfococcus* spp., *Desulfofrigus* spp.	[43]

To amplify archaea, a nested PCR approach was applied. The first PCR was run with primers Arch21f/Uni1392r (Table 1) under the condition described in Table S1 and Table S3. The nested PCR was run with the primers Arch21f-FAM and Arch958r (Table 1) using the product from the first PCR as a template under the condition described in Table S1 and Table S4. The products were assayed on a 1% agarose gel. Bands of the correct length on the gel were cut and purified using the QIAquick Gel Extraction Kit (Qiagen, Hilden, Germany). The PCR product (100 ng) obtained from gel extractions was digested by 2.5 units of restriction enzyme Hha1 at 37°C for 3 hours. The digested DNA fragments were then washed and dried according to the procedure as described above.

The resultant DNA fragments with fluorescent labels were analyzed at the Genetic Service Unit (University Hospital, Gent, Belgium). Statistical analysis of the patterns was performed using Bionumerics 5.1 software (Applied Maths, Sint-Martens-Latem, Belgium) [26].

Extracted data were processed for ecological interpretation in the following aspects:

1. Richness—the number of bands in one T-RFLP pattern was used an indicator of community richness.
2. Community organization (Gini)—calculated from the normalized area between a given Lorenz curve and the perfect evenness line [26]. This calculation yields a single value used to describe the degree of evenness of the community.
3. Similarity via depth—used to describe the rate of community changes with sediment depth using moving window analyses [26]. The value presented is the similarity of T-RFLP patterns of two samples from the sediment depths next to each other. For example, the T-RFLP pattern of sediment sample at 20 cm bsf was compared to that of sediment sample at 10 cm bsf, and the similarity is shown as a unit of percentage in y axil.

### Cell identification and quantification of the incubated samples

The mud breccia slurry samples from the incubation experiment were taken at days 0 and 176 and fixed in 4% formaldehyde (1 part slurry to 3 parts formaldehyde) overnight at 4°C. The fixed sample was then washed with PBS buffer twice and further diluted 2000 times with PBS. Next, 8 ml diluted slurry was filtered onto a circular GTTP polycarbonate filter (0.2 µm, Millipore, Germany) with a diameter of 2.5 cm. Cell staining and catalyzed reporter deposition fluorescence *in situ* hybridization (CARD-FISH) analysis were performed on the filter based on the protocol of Pernthaler et al [27]. DAPI (4′,6′-diamidino-2-phenylindol) staining was used to assess the total cell count. The probes used to identify ANME groups and SRB are listed in Table 1. Cells were counted under a microscope (Zeiss, Carl Zeiss Microimaging GmbH, Germany) with 50 fields of view (140 µm * 90 µm) used for each hybridization. The detection limit of this method was 2*10^5^ cell (aggregate)/ml raw sediment.

## Results

### Field observations and pore water profile

The sediment core from the Ginsburg MV reached 904 m of water depth and recovered 357 cm of sediment. When the core was cut open, voids, most likely from the decomposition of gas hydrates, were observed at depths of 53–58 cm, 111–113 cm, 128–133 cm, 173 cm, 193 cm, and 213–218 cm bsf. A thin layer consisting of a few mm of hemipelagic trapping was observed on the surface.

**Figure 2:**
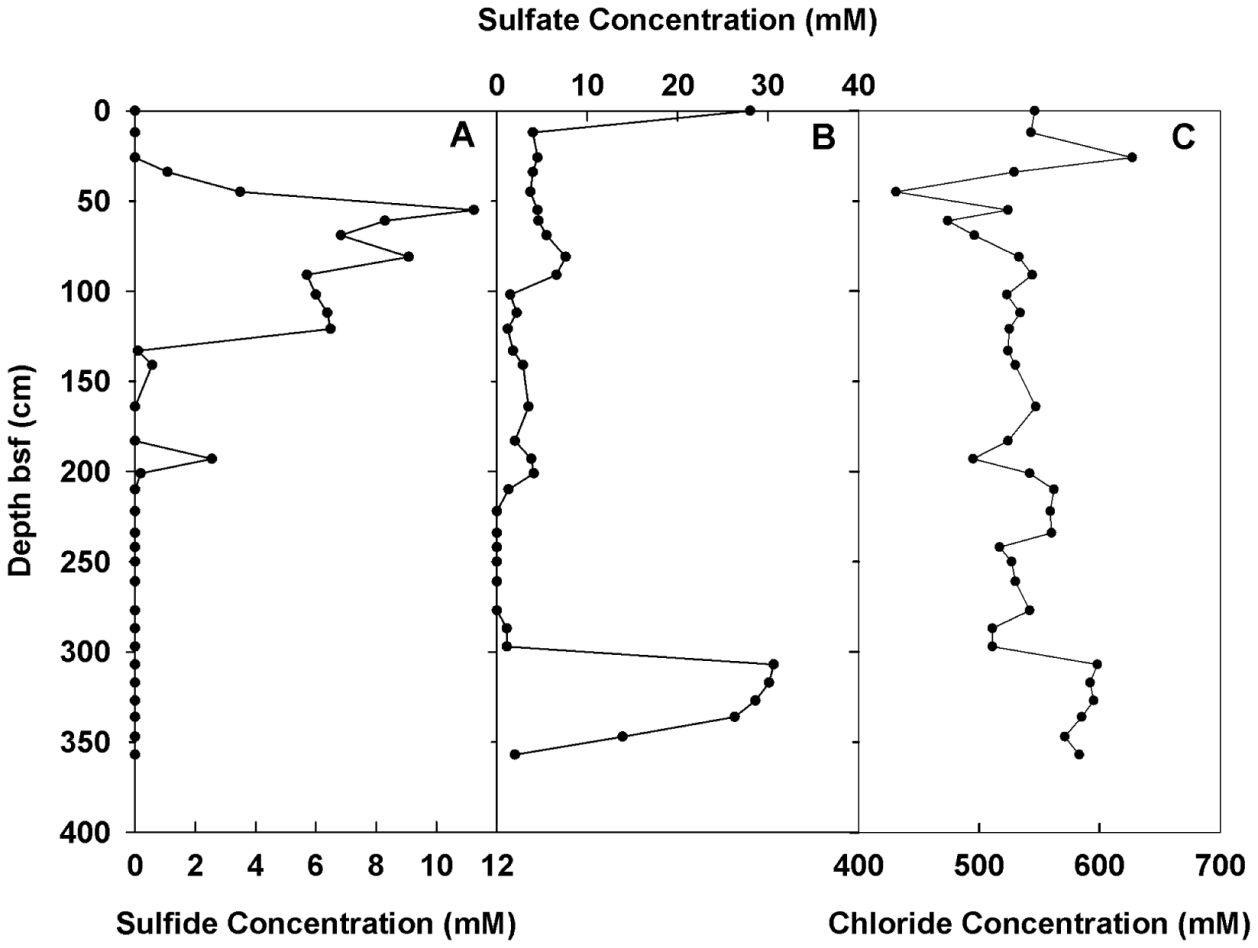
The concentrations of ions, including sulfide (A), sulfate (B) and chloride (C), in the pore water of the Ginsburg MV.

In the pore water, sulfide was detected at 34–141 cm bsf (maximum 11.2 mM) and at 193 cm bsf (maximum 2.6 mM) (Fig. 2A). The sulfate concentration decreased from ambient seawater levels (i.e., 28.0 mM) to 4.0 mM within the top 12 cm of sediment and remained stable until 307 cm bsf; below this depth, a sharp increase was observed (Fig. 2B). The seawater chloride concentration (544±2 mM) was measured in the top 12 cm of sediment. The chloride concentrations varied from 431 to 627 mM in the top 34 cm and remained relatively stable until 297 cm bsf, with an average value of 528±21 mM (Fig. 2C).

### *In vitro* SR-AOM activity at different depths

**Figure 3:**
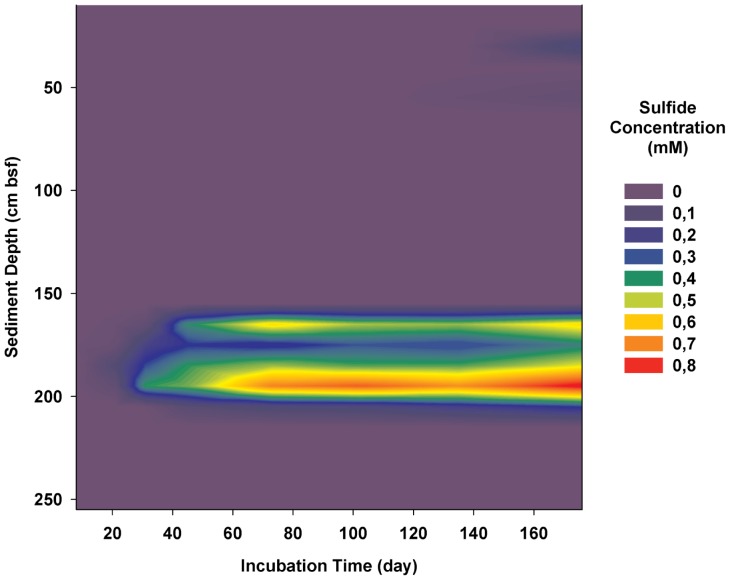
Cumulative sulfide concentrations at different sediment depths during 176 days of *in vitro* incubation with a methane and sulfate supply.

When methane and sulfate were supplied, sediments from different depths responded differently in terms of SR-AOM activities. Throughout the 176 days of the incubation period, two distinguishable active zones were formed. A shallow active zone was defined at 30–55 cm bsf in which the sediments only showed low SR activity after 102 days of incubation (Fig. 3). The overall SR activities during the 176-day incubation period were in the range of 0.3–1.4 nmol/ml rs/d. When the SR was calculated between day 102 (when sulfide production was observed) and day 176 (the end point of the incubation), the rates were in the range of 0.6–3.2 nmol/ml rs/d. No detectable AOM activity was observed in these samples.

**Figure 4:**
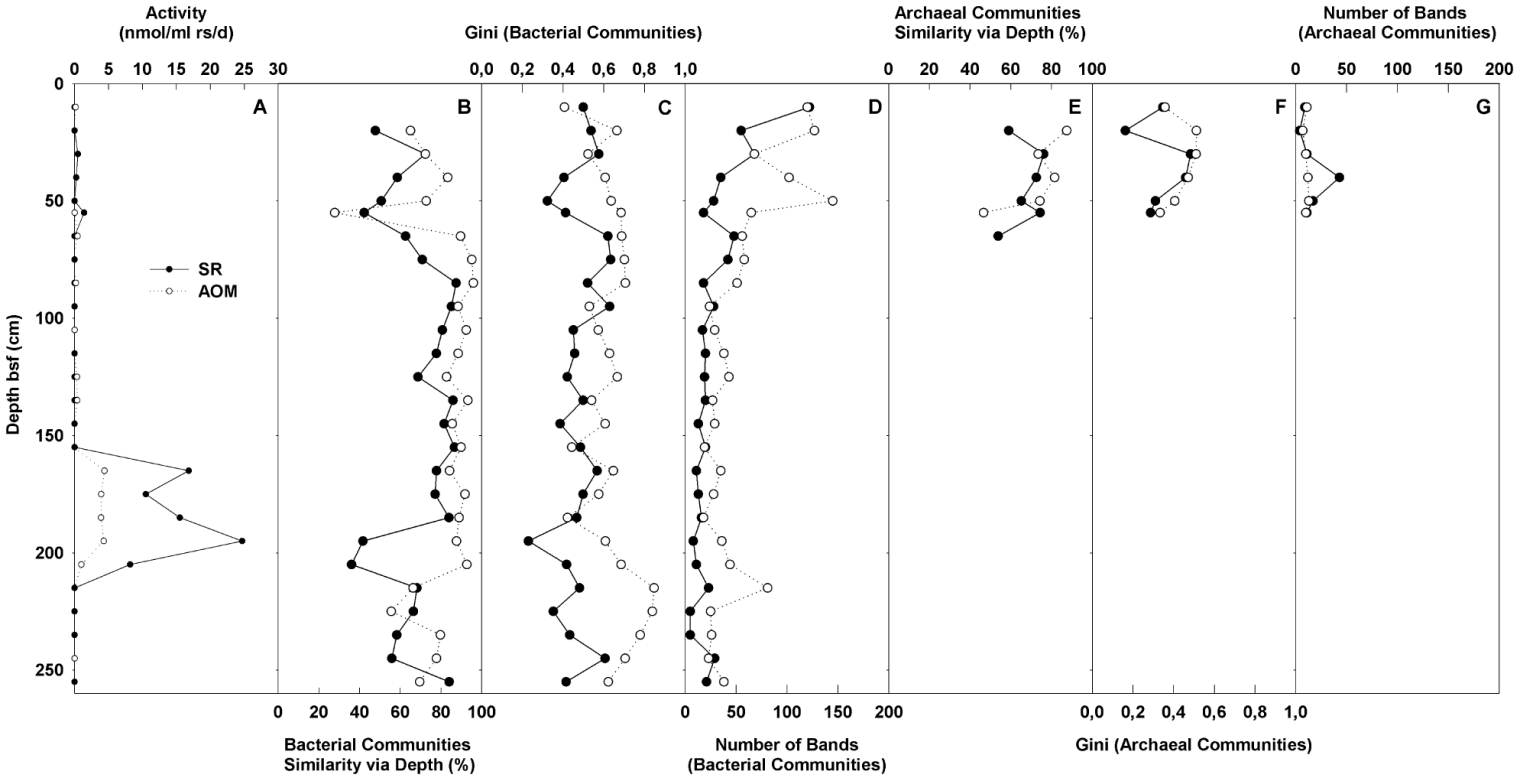
The microbial activity and community structures as an effect of 176 days of *in vitro* incubation. A) The overall SR/AOM activities; B/E) the bacterial/archaeal communities' similarity with respect to sediment depth; C/F) the bacterial/archaeal communities' evenness expressed as a Gini value; D/G) the bacterial/archaeal communities' richness. Legend for A: the solid dot is the SR rate, and the circle is the AOM rate. Legend for B-G: the solid dot is the value at day 0, and the circle is the value at day 176.

A deeper AOM active zone was defined at 165–205 cm bsf. In the presence of methane and sulfate, this interval exhibited an immediate production of sulfide, which remained active throughout the incubation experiment (Fig. 3). The highest SR activity, 24.7 nmol/ml rs/d, was detected at a depth of 195 cm bsf. This sediment layer also showed AOM activity, with rates of 1.4–6.2 nmol/ml rs/d (Fig. 4A). The remainder of the mud breccia section revealed no SR or AOM activities (Fig. 3).

### Community structure and dynamics

During the incubation experiment, diverse bacterial communities were described throughout the mud breccia sample at different sediment depths (Fig. 4B). Throughout the incubation period, the average bacterial communities' similarity via depth increased from 68% at day 0 to 81% at day 176. After incubation, the average bacterial communities' similarity via depth of a mud breccia sample covering the interval of 55 cm–205 cm bsf reached 90%. Fig. 4C shows that the uppermost 40 cm exhibited moderate bacterial evenness (Gini), and these values increased with increasing depth especially after incubation. In contrast, Fig. 4D shows that bacterial richness decreased with increasing depth within the top 75 cm both before and after incubations.

Archaeal T-RFLP patterns could only be obtained from the uppermost 65 cm of sediment because that concentrations of archaeal cells are below the detection limit of the method applied in the present work. Compared with bacterial communities, archaeal communities showed much lower richness (Fig. 4G).

### Cell quantification and identification

Cell counts based on DAPI staining demonstrated that the cell concentration in the shallow active zone (i.e., sediment from 55 cm bsf) was one log unit higher than that in the deeper active zone (i.e., sediment from 195 cm bsf) (Table 2). After incubation, a 70% decrease in total biomass was found at a sediment depth of 55 cm bsf. Here, the abundance of ANME-1 cells remained constant, and their relative concentration increased from 6% before to 20% after the incubation. The ANME-2 and SRB contents were just above the detection limit, and ANME-3 cells were not detected. In contrast, the incubation had no effect on total cell numbers in mud breccia at 195 cm bsf but clearly stimulated the growth of ANME-2 and SRB, especially in the form of aggregates (diameter 2–10 µm) (Table 2).

**Table 2:** Cell identification and quantification by CARD-FISH.

Prokaryotic group targeted	cell (aggregate)/ml	55 cm bsf		195 cm bsf	
		Before incubation	After incubation	Before incubation	After incubation
Total	Cell	1*10^8^	3*10^7^	8*10^6^	8*10^6^
	Aggregate	5*10^5^	6*10^5^	9*10^5^	2*10^6^
ANME-1	Cell	6*10^6^	6*10^6^	<2*10^5^	2*10^5^
	Aggregate	<2*10^5^	<2*10^5^	<2*10^5^	<2*10^5^
ANME-2	Cell	2*10^5^	4*10^5^	2*10^5^	<2*10^5^
	Aggregate	<2*10^5^	<2*10^5^	<2*10^5^	8*10^5^
ANME-3	Cell	<2*10^5^	<2*10^5^	<2*10^5^	<2*10^5^
	Aggregate	<2*10^5^	<2*10^5^	<2*10^5^	<2*10^5^
SRB	Cell	<2*10^5^	<2*10^5^	<2*10^5^	2*10^5^
	Aggregate	<2*10^5^	<2*10^5^	<2*10^5^	1*10^6^

## Discussion

### Multiple AOM active zones

The Ginsburg MV is located in the eastern part of Gulf of Cadiz (Fig. 1), an active MV and fluid-venting region [23], [28]. Mud breccia was collected from a relatively recent mudflow and it contained gas hydrates; the presence of gas hydrates is a known phenomenon for this MV [23]. For the last decade, the Ginsburg MV has been reported as an active structure, and its tectonic structure and geobiochemistry have been intensively studied by different programs and scientific groups [17], [21]–[23], [29]–[31]. In the present study, the sulfide and sulfate distribution profiles identifies the location of the SMTZ at a depth of 30–70 cm bsf (Fig. 2). This result is in agreement with published data on hydrocarbon gases, pore water parameters and AOM/SR rates measurements from the same MV [23], [31]. Meanwhile, Figs. 2B and 2C show that at a depth of ca. 190 cm bsf, the behavior of sulfide and sulfate curves indicates the possibility of an additional AOM active zone. The sulfate profile clearly suggests an alternative to the seawater sulfate source, the nature of which has thus far not been elucidated. The occurrence of specific void-like structures resulting from the dissociation of gas hydrates was also documented within the same mud breccia interval. Accordingly, pore water parameters suggest two potentially active AOM intervals within the uppermost 2.5 m of mud breccia.

The evidence of two separate active AOM zones is also supported by the *in vitro* incubation experiment in which additional methane and sulfate were supplied. Under these conditions, immediate and/or delayed SR was detected within similar sedimentary layers, i.e., at 30–55 cm bsf and at 165–205 cm bsf. Furthermore, despite the low sulfide concentrations in the pore water, mud breccia from the deep AOM zone showed immediate SR and AOM activity that was ten times higher than the activity in the AOM interval above (Fig. 4A). Therefore, the vertical distribution profiles of pore water form a valuable tool for targeting the potential AOM active zones but are not necessarily sufficient to quantify the rates of the process. Although measured *in vitro* activity is strongly affected by the incubation conditions, *in vitro* measurements remain a valuable indicator for understanding *in situ* microbial activity. We are aware that in this study, the incubation was performed as single microcosms without replicates due to the biomass limitation, which may cause bias. Still, the sediment depths with AOM activity were clustered into two intervals, which is strong evidence to locate the AOM active zones. The *in vitro* experiments led to the hypothesis that, in the presence of necessary electron donors and acceptors, anaerobic methanotrophy can be fuelled and sustained even at great sedimentary depths.

The discovery of multiple AOM active intervals in one sediment core suggests that deep AOM activity should not be overlooked in methane budget calculations. Based on the currently available data, the sources and sinks of oceanic methane are not balanced with the standing stock. For example, according to the estimated data from Reeburgh [10], the reciprocal of the measured and modeled specific turnover rates for the deep ocean (0.01–0.02 year^−1^) provides a residence time based on a removal time of 50–100 years. In contrast, dividing the open ocean standing stock (43.2 Tg) by estimated methane fluxes from MVs (27 Tg year^−1^) and shelf additions (20 Tg year^−1^) yields an estimated residence time of between 2 and 3 years based on the addition of methane. These data on methane turnover rate to calculate the budget were often generated from *in situ* or *in vitro* radioactive tracer incubations, which is a sensitive method but is restricted to surface sediment and short-term monitoring. Deep or delayed methanotrophic processes have therefore been largely ignored. Based on the experimental data in this study, the deep layer AOM activity is one order of magnitude higher than that in SMTZ. This deep buried methane sink could at least partially reconcile the gap of current methane budget.

### Response of ANMEs to methane and sulfate supply

Although ANME lipid biomarkers, especially ANME-1, appeared to be present in every interval of the Ginsburg MV core down to 180 cm bsf [23], both the pore water profile and our incubation results lead to the conclusion that AOM activity is only present at certain sediment depths. It is not surprising that sediments all along the sampling core might have been exposed to a methane- and sulfate-rich environment during certain historical periods because the discharge of hydrocarbon-rich fluid is a common phenomenon in the Gulf of Cadiz and the Ginsburg MV is characterized as an active structure with extensive mud diapirism and mud volcanism. The relatively broad distribution of ANME lipids along the MV deposits indirectly indicates methane flow and, thus, a migration or displacement of the SMTZ in the Ginsburg MV [23]. The AOM microbial activity in recent or ancient SMTZs cannot be always restored within a six month period simply by providing fresh methane and sulfate in laboratory conditions, implying that the *in situ* development of the AOM community after methane- and sulfate-rich fluid migration is a long-term process.

The incubation conditions in this study apparently favor the activity and growth of ANME-2 over ANME-1. An increase in ANME-2 cells and aggregates has been observed in both of the AOM active zones. Moreover, sediment from the deeper location, in which ANME-2 is dominant, has higher SR-AOM activity compared with that from the SMTZ, where ANME-1 is dominant. In fact, in addition to ANME-2, ANME-1 has been selectively enriched in the SMTZ; the ANME-1 cell numbers remained constant, whereas there was a 70% decay of total free-living cells (counted by DAPI staining, Table 2). Alternatively, this 70% decay of cells might have contributed to heterotrophic SR and other microbial processes; however, we did not measure such processes. This incubation result is in agreement with investigations from other researchers regarding the niche differentiation of ANME-1 and ANME-2. For example, it has been suggested that compared to ANME-1, ANME-2 is dominant in environments with higher SR activity, lower flow rates, relatively elevated methane partial pressures and temperatures in the range of 10–15°C [32]–[34]; such a pattern is similar to what we observed in our incubations, especially in the deeper active zone.

### Community response to *in vitro* incubations

The incubation promoted a tendency for prokaryotic communities, especially bacterial communities, at different depths to converge (Figs. 4B and 4E). Concurrently, the bacterial community richness also rose as a result of the incubation, even in sediment intervals without detectable SR activity (Fig. 4D). The entire incubation period lasted 176 days, at which point there was almost no overpressure inside certain incubation bottles; methane gas had been consumed by microorganisms and lost during sampling. During the incubation period, the ANME and SRB cells reproduced 1–3 times (calculated from the data in Table 2). Due to the extremely low growth rates of ANME and SRB (with doubling times of approximately 2 months), they are not major contributors in terms of abundance to overall prokaryotic community dynamics despite the fact that they are key players in primary production in the cold seep ecosystem. The rapid changes in community structure and the increase in richness are thought to be driven by the increase of substrate diversity during incubation [35]. Initially, methane and CO_2_ were the only sources of additional carbon; later, the metabolic products of ANME and SRB and the decay of biomass allowed more complex carbon compounds to become available to the system. It must be taken into account that, due to the low biomass content from our samples, this T-RFLP analysis is unlikely to capture rare species. It has been proven that when a preconditioned community colonizes a familiar habitat, the community structure is more predictable [36]. It is therefore reasonable to believe that the species that were observed to increase using T-RFLP were also historically dominant. The increase of biodiversity suggests that a more complex metabolic network within local ecosystems was stimulated due to the incubation, whereas the increase in similarity via depth suggests that the metabolic networks at different sediment depths share similar groups of microbes. Future work with higher efficiency and sequencing data, which may be accomplished by using pyrotags, could detail such communities and their functions.

## Concluding Remarks

In this study, we attempted to identify how the prokaryotic community would respond to changes in methane and sulfate intensities in Ginsburg MV sediment. After a long-term *in vitro* incubation, a deeply buried AOM active zone was discovered besides the SMTZ, where the AOM activity was one order of magnitude higher than that of SMTZ. This discovery calls up our attention that the potential AOM activity at deep subsurface even below SMTZ should not be ignored or underestimated in oceanic methane budget calculation. Moreover, the incubation condition, a highly reduced environment with high sulfate and methane concentration but no flux nor organic nutrient supply, caused a selective enrichment of ANME (especially ANME-2 rather than ANME-1) and SRB. Although ANME-2 and SRB are the main contributors to SR-AOM activity, bacteria, who may be living on the organic compounds released from cellular metabolism and decay, are taking the major part to shape the overall community structure. This study provides direct information regarding to the spatial distribution and activity of archaea and bacteria in cold seep environments.

## Supporting Information

Table S1 **Reagents concentrations for bacterial and archaeal PCR using KAPA2G Robust PCR kit (KAPA Biosystems, Wilmington, USA).**
(DOCX)

Table S2 **Thermal protocol for bacterial PCR using primer set 27f-FAM/907r.**
(DOCX)

Table S3 **Thermal protocol for archaeal PCR using primer set Arch21f/Uni1392r.**
(DOCX)

Table S4 **Thermal protocol for archaeal PCR using primer set Arch21f-FAM/Arch958r.**
(DOCX)
