# Correction: Stratified Community Responses to Methane and Sulfate Supplies in Mud Volcano Deposits: Insights from an *In Vitro* Experiment

**DOI:** 10.1371/journal.pone.0117121

**Published:** 2015-01-26

**Authors:** The PLOS ONE Staff

There is an error in affiliation 3 for author Alina Stadnitskaia. Affiliation 3 should be: Department of Marine Organic Biogeochemistry, Royal Netherlands Institute for Sea Research, Texel, the Netherlands.
