# Supplementary material for: Stratified Community Responses to Methane and Sulfate Supplies in Mud Volcano Deposits: Insights from an In Vitro Experiment
Source: PLoS One. 2014 Nov 13;9(11):e113004. doi: 10.1371/journal.pone.0113004 (PMC4231134; doi:10.1371/journal.pone.0113004)
Supplement: Table S1 — Reagents concentrations for bacterial and archaeal PCR using KAPA2G Robust PCR kit (KAPA Biosystems, Wilmington, USA). (DOCX) [file pone.0113004.s001.docx]

Table S1 Reagents concentrations for bacterial and archaeal PCR using KAPA2G Robust PCR kit (KAPA Biosystems, Wilmington, USA)

| **Reagent** | **Volume μl** |
| --- | --- |
| Bacterial PCR | |
| PCR water | 12.9 |
| Buffer B + Mg^2+^ | 5 |
| dNTP | 0.5 |
| Taq polymerase | 0.1 |
| Primer 1 (50μM) | 0.25 |
| Primer 2 (50μM) | 0.25 |
| Enhancer 1 | 5 |
| Template | 1 |
| Total | 25 |
| Archaeal PCR | |
| PCR water | 17.9 |
| Buffer A + Mg^2+^ | 5 |
| dNTP | 0.5 |
| Taq polymerase | 0.1 |
| Primer 1 (50μM) | 0.25 |
| Primer 2 (50μM) | 0.25 |
| Template | 1 |
| Total | 25 |
