# Supplementary material for: Stratified Community Responses to Methane and Sulfate Supplies in Mud Volcano Deposits: Insights from an In Vitro Experiment
Source: PLoS One. 2014 Nov 13;9(11):e113004. doi: 10.1371/journal.pone.0113004 (PMC4231134; doi:10.1371/journal.pone.0113004)
Supplement: Table S2 — Thermal protocol for bacterial PCR using primer set 27f-FAM/907r. (DOCX) [file pone.0113004.s002.docx]

Table S2 Thermal protocol for bacterial PCR using primer set 27f-FAM/907r

| **Step** | **Condition** | **Cycles** |
| --- | --- | --- |
| 1. Initial denaturation | 95 °C, 5min |  |
| 2. Denaturation | 94 °C, 1min | 33 |
| 3. Primer annealing | 52 °C, 1min |  |
| 4. DNA-synthesis | 72 °C, 3min |  |
| 5. Final extension | 72 °C, 10min |  |
| 6. Storage of the product inside instrument | 4 °C, +∞ |  |
