# Supplementary material for: Stratified Community Responses to Methane and Sulfate Supplies in Mud Volcano Deposits: Insights from an In Vitro Experiment
Source: PLoS One. 2014 Nov 13;9(11):e113004. doi: 10.1371/journal.pone.0113004 (PMC4231134; doi:10.1371/journal.pone.0113004)
Supplement: Table S3 — Thermal protocol for archaeal PCR using primer set Arch21f/Uni1392r. (DOCX) [file pone.0113004.s003.docx]

Table S3 Thermal protocol for archaeal PCR using primer set Arch21f/Uni1392r

| **Step** | **Condition** | **Cycles** |
| --- | --- | --- |
| 1. Initial denaturation | 95 °C, 5min |  |
| 2. Denaturation | 94 °C, 1min | 10+20* |
| 3. Primer annealing | 65-60 °C, 1min |  |
| 4. DNA-synthesis | 72 °C, 3min |  |
| 5. Final extension | 72 °C, 10min |  |
| 6. Storage of the product inside instrument | 4 °C, +∞ |  |

* Using a touchdown program: the annealing temperature decreased from 65°C to 60°C at 0.5°C intervals every cycle for 10 cycles and at a 60°C annealing temperature for an additional 20 cycles.
