# Supplementary material for: Stratified Community Responses to Methane and Sulfate Supplies in Mud Volcano Deposits: Insights from an In Vitro Experiment
Source: PLoS One. 2014 Nov 13;9(11):e113004. doi: 10.1371/journal.pone.0113004 (PMC4231134; doi:10.1371/journal.pone.0113004)
Supplement: Table S4 — Thermal protocol for archaeal PCR using primer set Arch21f-FAM/Arch958r. (DOCX) [file pone.0113004.s004.docx]

Table S4 Thermal protocol for archaeal PCR using primer set Arch21f-FAM/Arch958r

| **Step** | **Condition** | **Cycles** |
| --- | --- | --- |
| 1. Initial denaturation | 95 °C, 5min |  |
| 2. Denaturation | 94 °C, 1min | 20 or 30* |
| 3. Primer annealing | 62 °C, 1min |  |
| 4. DNA-synthesis | 72 °C, 3min |  |
| 5. Final extension | 72 °C, 10min |  |
| 6. Storage of the product inside instrument | 4 °C, +∞ |  |

* For samples that did not yield bands at the correct length after 20 cycles of amplification in the nested PCR, the nested PCR was repeated increasing the number of cycles of amplification up to 30. Samples that did not yield clear bands at the correct length after 30 cycles of nested PCR were considered to have archaea contents below our method’s threshold of detection.
